# Supplementary material for: The interactome of intact mitochondria by cross-linking mass spectrometry provides evidence for coexisting respiratory supercomplexes
Source: Mol Cell Proteomics. 2017 Dec 8;17(2):216–32. doi: 10.1074/mcp.RA117.000470 (PMC5795388; doi:10.1074/mcp.RA117.000470)
Supplement: Supplemental Data [file supp_RA117.000470_133922_0_supp_23978_fzffwf.zip › spectra_annotation/mito_spectra_annotation/380-1-6-1-2-1.pdf]

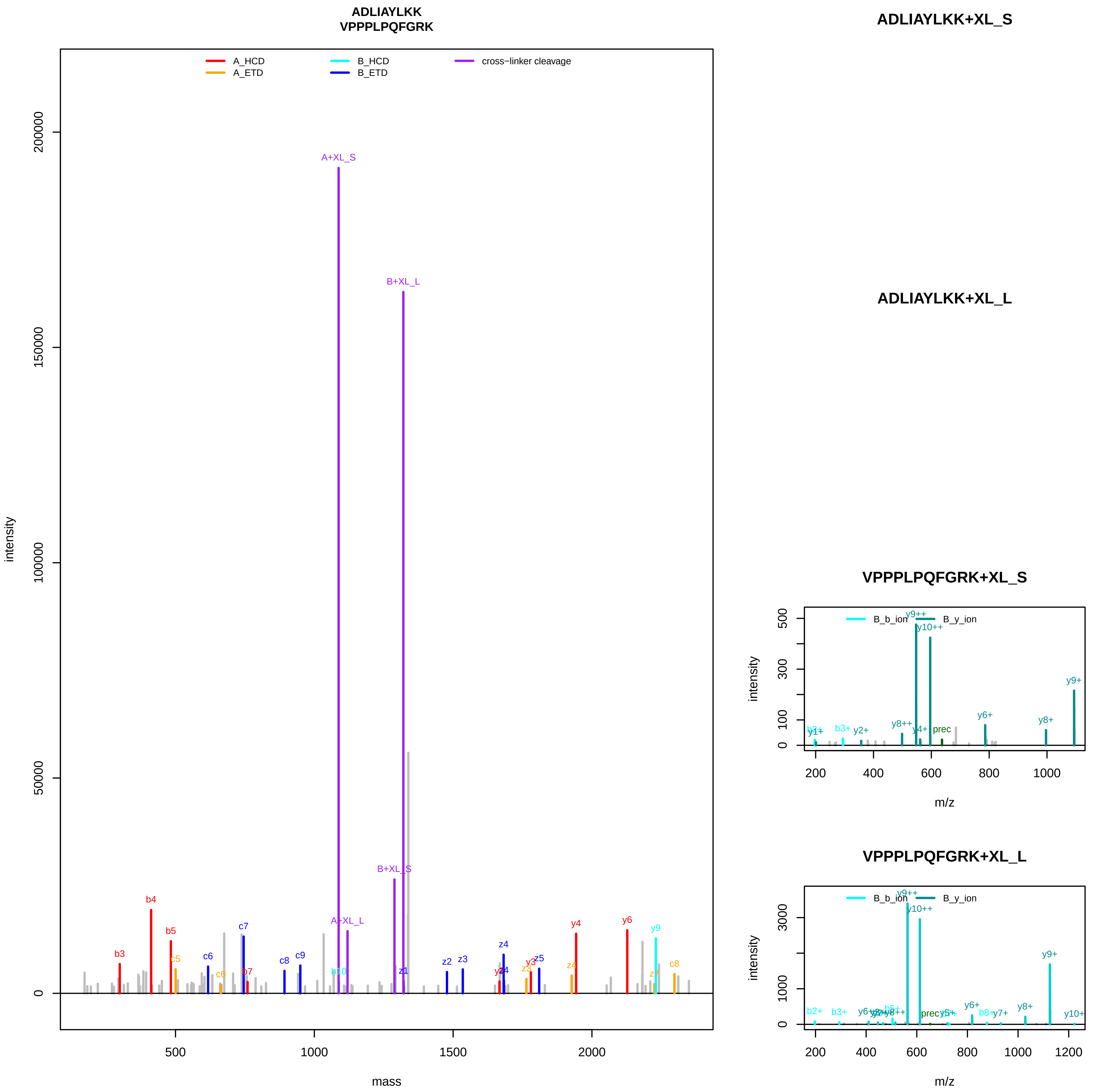
ADLIAYLKK
VPPPLPQFGRK
A_HCD
A_ETD
B_HCD
B_ETD
cross−linker cleavage
A+XL_S
B+XL_L
B+XL_S
A+XL_L
b3
b4
b5
c5
c6
c7
b7
c8
c9
b10
z1
z2
z3
z4
y3
z5
y4
y6
y9
c8
intensity
mass
ADLIAYLKK+XL_S
ADLIAYLKK+XL_L
VPPPLPQFGRK+XL_S
B_b_ion
B_y_ion
y9++
y10++
y9+
y6+
y8+
y8++
y4+
prec
b3+
y2+
y1+
m/z
VPPPLPQFGRK+XL_L
b2+
y7+
y10+
